# Increased Eicosanoid Levels in the Sugen/Chronic Hypoxia Model of Severe Pulmonary Hypertension

**DOI:** 10.1371/journal.pone.0120157

**Published:** 2015-03-18

**Authors:** Aysar Al-Husseini, Dayanjan S. Wijesinghe, Laszlo Farkas, Donatas Kraskauskas, Jennifer I. Drake, Ben Van Tassel, Antonio Abbate, Charles E. Chalfant, Norbert F. Voelkel

**Affiliations:** 1 Pulmonary and Critical care Medicine Division, Victoria Johnson Center for Lung Research, Richmond, Virginia, United States of America; 2 Department of Internal Medicine, Montefiore Mount Vernon Hospital, Westchester, New York, United States of America; 3 Department of Biochemistry and Molecular Biology, Virginia Commonwealth University-School of Medicine, Richmond, Virginia, United States of America; 4 Division of Cardiology, Virginia Commonwealth University, Richmond, Virginia, United States of America; 5 Hunter Holmes McGuire Veterans Administration Medical Center, Richmond, Virginia, United States of America; 6 The Massey Cancer Center, Richmond, Virginia, United States of America; 7 Virginia Commonwealth University Reanimation Engineering Science Center (VCURES), Richmond, Virginia, United States of America; University of Illinois College of Medicine, UNITED STATES

## Abstract

Inflammation and altered immunity are recognized components of severe pulmonary arterial hypertension in human patients and in animal models of PAH. While eicosanoid metabolites of cyclooxygenase and lipoxygenase pathways have been identified in the lungs from pulmonary hypertensive animals their role in the pathogenesis of severe angioobliterative PAH has not been examined. Here we investigated whether a cyclooxygenase-2 (COX-2) inhibitor or diethylcarbamazine (DEC), that is known for its 5-lipoxygenase inhibiting and antioxidant actions, modify the development of PAH in the Sugen 5416/hypoxia (SuHx) rat model. The COX-2 inhibitor SC-58125 had little effect on the right ventricular pressure and did not prevent the development of pulmonary angioobliteration. In contrast, DEC blunted the muscularization of pulmonary arterioles and reduced the number of fully obliterated lung vessels. DEC treatment of SuHx rats, after the lung vascular disease had been established, reduced the degree of PAH, the number of obliterated arterioles and the degree of perivascular inflammation. We conclude that the non-specific anti-inflammatory drug DEC affects developing PAH and is partially effective once angioobliterative PAH has been established.

## Introduction

Inflammatory cells have been thought to contribute to the pathobiology of pulmonary hypertension (PH), ever since mast cell-derived histamine was considered as a mediator of hypoxic pulmonary vasoconstriction more than 40 years ago [1,2]. An early focus in the area of pulmonary hypertension and inflammation research was on arachidonic acid metabolites produced by inflammatory cells and endothelial cells. Early on eicosanoid metabolites had been measured in human forms of pulmonary hypertension [3], including neonatal pulmonary hypertension [4]. Recent clinical trials examined the effects of low-dose aspirin treatment in patients with idiopathic pulmonary hypertension[5,6] with the therapeutic goal to reduce thromboxane synthesis [5], while chronic infusion of prostacyclin remains an expensive treatment of patients with severe PAH; this treatment improves survival of many patients [7] Yet still today relatively few studies have experimentally addressed whether lipid metabolites cause or modulate pulmonary hypertension [8,9,10,11,12,13] and the published data reflect somewhat inconsistent results.

We have previously characterized a rat model of severe PAH [14,15,16] which shares several important features of human forms of severe PAH, including a lumen-obliterating pulmonary angiopathy and inflammation and right heart failure; we hypothesized that eicosanoid metabolites would be elevated in the inflamed lung tissues from pulmonary hypertensive animals.

Our first goal was to show that the enzymes which are of critical importance for arachidonic acid metabolism: cytosolic phospholipase A_2_ (cPLA_2_) and cyclooxygenase 2 (COX-2) are highly expressed in the lungs from severe pulmonary hypertensive rats. To achieve this goal we used Western blot analysis and we localized 5-lipoxygenase and leukotriene hydrolase (LTA_4_) in the lung vascular lesions using immunohistochemistry. In addition, we measured the lung tissue concentration of a large number of arachidonic acid-derived metabolites, by mass spectroscopy.

Because the cells which make up the lumen-obliterating lesions in the lungs from PAH patients are abnormal and have been characterized as ‘quasi malignant’ [17] and because of the cellular and molecular cross talk between chronic inflammation, angiogenesis and cancer and a postulated role for cyclooxygenase 2 (COX-2) metabolites, in particular prostaglandin E_2_, in the pathobiology of metastasizing cancers [18,19,20,21,22], our second goal was to test a COX-2 inhibitor in the SuHx model of severe angioobliterative pulmonary hypertension (PAH)[16,23,24].

A few studies have previously addressed the role of COX-2 in mouse models of pulmonary hypertension [25,26,27]. In addition, Delannoy et al [28] reported in mice that chronic hypoxia caused a COX-2 dependent hyperactivity of the pulmonary arteries isolated from these animals; this was associated with increased production of 8-iso-PGF2α, a marker of oxidative stress [29]. However, Seta et al reported that oxidative stress was increased in COX-2 knockdown mice with monocrotaline-induced PAH [25]. In other studies it has been shown that naïve homozygous COX-2-null mice did not have PH, but developed higher right ventricular systolic pressure (RVSP) when exposed to hypoxia for 2 weeks and that the pulmonary arterioles of these mice showed a greater degree of muscularization when compared with the WT mice [27].

We now show that the COX-2 inhibitor SC-58125 [30] affected the eicosanoid metabolite profile differently in the lungs from the SuHx pulmonary animals when compared to the right ventricle (RV) tissue samples and surprisingly that chronic COX-2 inhibition did not worsen the PAH in this model.

Because the COX-2 inhibitor SC-58125 tended to reduce the lung tissue levels of cysteinyl leukotrienes C4 and D4 and because 5-Lipoxygenase (5-LO) inhibitors had already been shown to reduce PH in the chronic hypoxia and monochrotaline models [11,13], we tested whether diethylcarbamazine [11] an inexpensive antihelminthic drug used in tropical zones to treat filariasis and a 5-LO inhibitor, would prevent or ameliorate PAH in the SuHx rat model.

Our preclinical studies demonstrate elevated eicosanoid levels in the lung and heart tissue samples from rats subjected to the SuHx protocol and that treatment with a COX-2 inhibitor did not worsen the PAH, while diethylcarbamazine impacted the pulmonary vascular disease in this model of severe PAH.

## Material and Method

### Animal Models

All experiments were approved by the Institutional Animal Care and Use Committee of Virginia Commonwealth University. Pulmonary hypertension was induced in male Sprague-Dawley rats (250 g BW) as follows: the animals received a single s.c. injection of the VEGF receptor tyrosine kinase inhibitor (Sugen 5416, 20mg/kg) and were exposed to chronic hypoxia (SuHx model), as described previously [14,31]. Age-matched and gender-matched rats were exposed to 10% hypoxia for 3 weeks in the prevention studies (n = 4 in SC-58125 experiment & n = 8 in Diethylcarbamazine experiment), and for 4 weeks followed by a return to room air for 2 weeks in the intervention studies (n = 8). Control animals were placed in room air for the same period of time for each group (n = 4). In the prevention studies, SC-58125 (10 mg/kg; Cayman Chemical, Ann Arbor, MI) and Diethylcarbamazine (50 mg/kg; Sigma Aldrich) were dissolved in normal saline and administered intraperitoneally every other day for 21 days (n = 4 in SC-58125 experiment & n = 6 in Diethylcarbamazine experiment). In the intervention trial, Diethylcarbamazine (50 mg/kg) was given for 2 weeks of 10 doses in total. At the end of the exposure period each rat was anesthetized with an intramuscular injection of ketamine/xylazine. Animals which had undergone the intervention trial were subjected for echocardiograph study, for measuring diastolic right ventricular internal diameter. The thoracic cavities were opened by midline incision, and hemodynamic measurements, using a 4.5-mm conductance catheter (Millar Instruments, Houston, TX) and the Powerlab data acquisition system (AD Instruments, Colorado Springs, CO), were performed as described previously [31]. The right lung was removed, and frozen in liquid nitrogen. The left lung was inflated with 0.5% low-melting agarose at a constant pressure of 25cm H2O, fixed in 10% formalin for 48 hours and used for small pulmonary artery and IHC analysis. Right ventricular hypertrophy was measured as a ratio of right ventricular weight to left ventricular plus septal weight (RV/LV+S).

### Antibodies

We used the following antibodies: Rabbit anti-cPLA2, rabbit anti-COX-2, rabbit anti-COX-1 (Cell Signaling Technology, Inc., Beverly, MA), mouse anti-ß-actin (Sigma, St. Louis, MO), rabbit anti von Willebrand factor (Dako, Carpinteria, CA), rabbit anti 5-Lipoxygenase (5-LO) (Cell Signaling) and rabbit anti Leukotriene A4 hydroxylase (LTA4H) (LifeSpan Biosciences, Inc., Seattle, WA).

### Western blot analysis

Whole cell lysate from one lobe of the right lung was prepared using RIPA (Radio-Immunoprecipitation Assay) buffer (Sigma, St. Louis, MO) and the protein concentration was determined using BioRad Protein DC Protein Assay (BioRad, Hercules, CA). Whole cellular protein, (30 microgram per lane) was separated by SDS-PAGE with a 4–12% Bis-Tris Nupage gel (MES SDS running buffer) and blotted onto a PVDF membrane. The membrane was incubated with blocking buffer (5% nonfat dry milk/PBS 0.1% Tween 20) at room temperature for 1 hour. The membrane was then probed with the primary antibodies diluted in blocking buffer overnight at 4°C. Subsequently, membranes were incubated with horseradish peroxidase-conjugated anti-mouse or anti-rabbit antibody diluted 1:500 or 1:1000 respectively in blocking buffer. Blots were developed with ECL (PerkinElmer, Waltham, MA) on GeneMate Blue Basic Autorad Films (BioExpress, Kaysville, UT). Blots were scanned and densitometry analysis was done with ImageJ (National Institutes of Health 1997–2011, Bethesda, MD; http://imagej.nih.gov/ij).

### Histology and microscopy

Formalin fixed paraffin embedded lung sections (4μm) were used for staining Elastica van Gieson (EvG) (sigma) was stained according to the manufacture’s protocol. Von Willebrand Factor (vWF) immunohistochemistry was performed as previously published [32]. Immunofluorescence studies of 5-LO (1:25) and LTA4H (1:50) were performed according to the protocol previously published [32]. Images were taken with AxioImager AX10, Axiocam MRm and Axiovision 3.1 software (Carl Zeiss, Göttingen, Germany) for the vWF and Elastica Van Gieson. Studies for immunofluorescence of 5-LO and LTA4H, optical sections were acquired by laser-scanning confocal microscopy with a Leica TCS-SP2 confocal microscope and images were arranged with ImageJ. The confocal microscopy was performed at the VCU Department of Anatomy and Neurobiology Microscopy Facility, supported, in part, by funding from a NIH-NINDS Center core grant (5P30NS047463-02).

### Assessment of angioproliferative vascular lesions, media wall thickness and perivascular inflammation

A quantitative analysis of luminal obstruction was performed by counting at least 200 small pulmonary arteries (External diameter, <50 um) per lung section from each rat in the 2 groups by two investigators blinded to the treatment group. Vessels were assessed to grade for angioobliteration: no evidence of angioproliferation (open); partially obliterated (<50%); and full-luminal occlusion (obliterated) from two random left lung slices using vWF immunohistochemistry staining. For assessment of the media wall thickness (MWT), external diameter (ED) and MWT were measured of 30–40 pulmonary arteries, using Elastic Van Gieson stained sections in 2 orthogonal directions using AxioVision 3.1 software. ED was defined as the distance between external elastic lamina, while MWT was determined as the distance between external and internal elastic laminas. Vessels were categorized as follows: 25 < ED < 50 μm and 50 ≤ ED < 100 μm. MWT was calculated using the following formula: MWT (%) = (2 × MWT/ED) × 100%, as described previously [33]

For the purpose of assessing perivascular inflammation, fields were selected as described for determination of number of obliterated vessels. The perivascular infiltrate surrounding each pulmonary artery was quantified as 0: absent, 1: minimal with a single layer clustering of inflammatory cells; 2: moderate, with localized clustering of inflammatory cells; and, 3: abundant, with large clusters of inflammatory cells extending from the perivascular region towards adjacent alveoli as described previously by Stacher et al [34]. The final inflammatory score was the result of: [0 x n vessels with 0 score + 1 x n vessels with 1 score, 2 x n vessels with 2 score + 3 x n vessels with 3 score]/number of analyzed vessels. 100±36 vascular profiles were examined per lung.

## Mass Spectroscopy

Eicosanoids were analyzed in rat lung and right ventricle tissues as follows. Frozen tissues were thawed on ice and homogenized using an Omni TH tissue homogenizer to obtain a 10% (w/v) solution in PBS. The tissue homogenate (200 μl) was diluted with 1ml of LCMS grade ethanol containing 0.05% BHT and the samples were spiked with 10 ng of each internal standard. The internal standards used were, (*d*_4_) 6k PGF_1α_, [The stable metabolite of prostacyclin] (*d*_4_) PGF_2α_, (*d*_4_) PGE_2_, (*d*_4_) PGD_2_, (*d*_*4*_) LTB_4_, (d4) TXB_2_ [The stable metabolite of TXA2], (*d*_*4*_) LTC_4_, (*d*_*5*_) LTD_4_, (*d*_*5*_) LTE_4_, (*d*_8_) 5-hydroxyeicosatetranoic acid (5HETE), (*d*_8_) 15-hydroxyeicosatetranoic acid (15HETE), (*d*_*8*_) 14,15 epoxyeicosatrienoic acid, (*d*_8_) Arachidonic Acid, and (*d5*) Eicosapentaenoic acid. The samples were mixed using a bath sonicator followed by incubation for 5 hours in the dark at 4°C with periodic mixing via bath sonication. Following incubation, the insoluble fraction was precipitated by centrifuging at 6000g for 20 minutes and the supernatant was transferred into a new glass tube. The extracts thus obtained were dried under vacuum and reconstituted in 100 μl of LCMS grade 50:50 EtOH: dH2O for eicosanoid quantitation via UPLC ESI-MS/MS analysis. A 12 minute reversed-phase LC method utilizing a Kinetex C18 column (100 x 2.1mm, 1.7μm) and a Shimadzu UPLC was used to separate the eicosanoids at a flow rate of 500μl/min at 50°C. The column was first equilibrated with 100% Solvent A [acetonitrile: water: formic acid (20:80:0.02, v/v/v)] for two minutes and then 10 μl of sample was injected. 100% Solvent A was used for the first minute of elution. Solvent B [acetonitrile: isopropanol (20:80, v/v)] was increased in a linear gradient to 25% Solvent B to 2 minutes, to 45% until 5 minutes, to 60% until 7 minutes, to 75% until 8 minutes, and to 100% until 10 minutes. 100% Solvent B was held until 11 minutes, then was decreased to 0% in a linear gradient until 26 minutes, and then held until 30 minutes. The eicosanoids were then analyzed using a hybrid triple quadrapole linear ion trap mass spectrometer (ABSciex 5500 QTRAP,) via multiple-reaction monitoring in negative-ion mode. Eicosanoids were monitored using species specific precursor → product MRM pairs. The mass spectrometer parameters used were: curtain gas: 30; CAD: High; ion spray voltage: −3500V; temperature: 500°C; Gas 1: 40; Gas 2: 60; declustering potential, collision energy, and cell exit potential vary per transition.

### Statistical analysis

Data are presented as mean ± SEM. Two groups were compared with 2-tailed unpaired Student’s *t* test and more than 2 groups with 1-way ANOVA followed by Neuman-Keuls multiple comparison test. Statistical tests and graphs were done with GraphPad Prism 5.0 (GraphPad Software). P < 0.05 was considered significant.

## Results

### Effect of the COX-2 specific inhibitor SC-58125 on pulmonary hypertension, right ventricular hypertrophy & lung vascular remodeling

**Fig 1:**
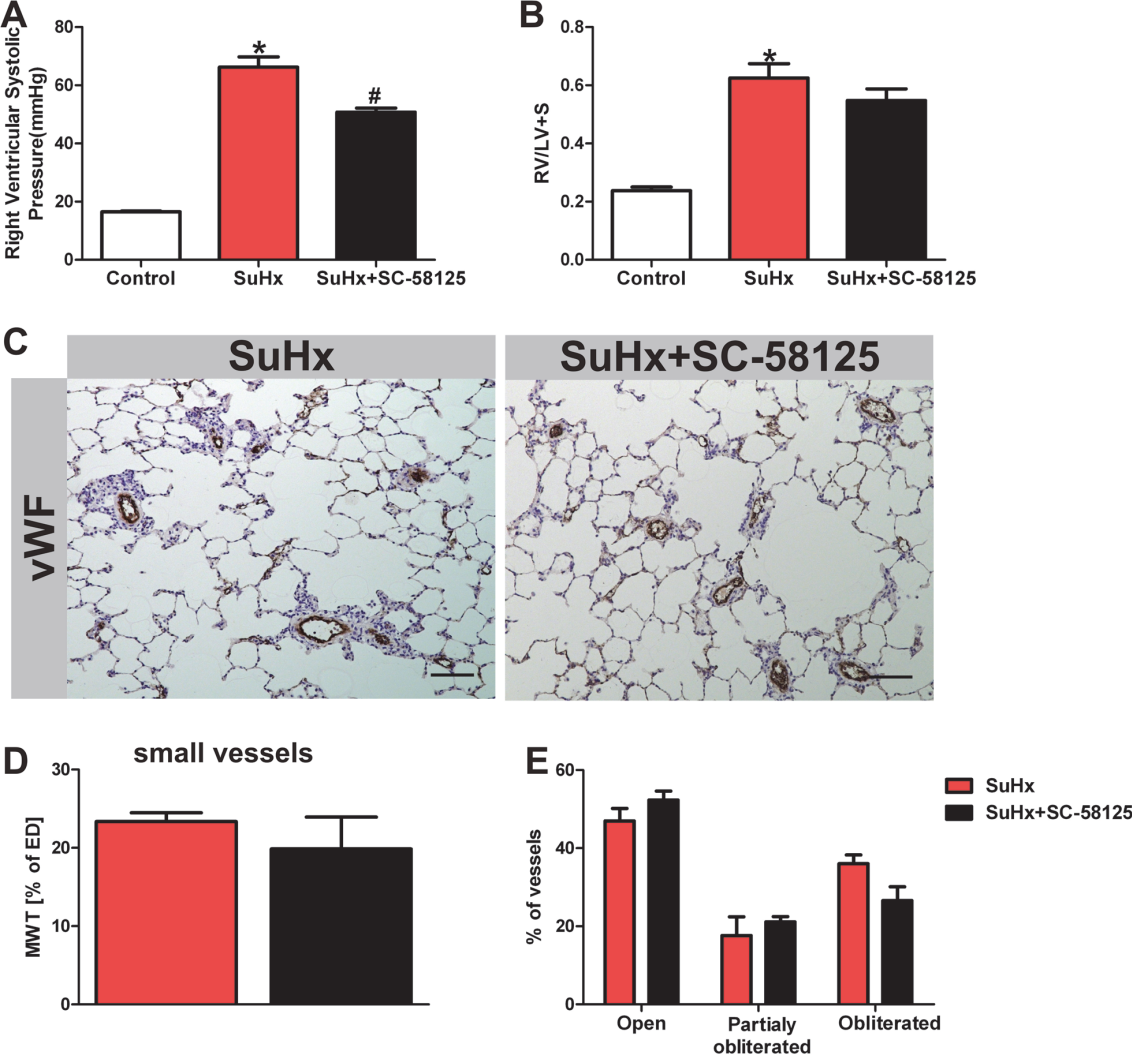
Effects of SC-58125 on hemodynamics, pulmonary artery muscularization and angioobliteration. The right ventricular systolic pressure measured (using a Millar catheter) is reduced in the SuHx rats following 3 weeks of treatment of the animals with the COX-2 inhibitor (A) (n = 4). There is no significant reduction of the right ventricular hypertrophy (RV/LV+S) (B) (n = 4). (C) Scale bar = 100μm. There is no significant reduction of the angioobliteration neither the muscularization in the lungs from rats treated with the inhibitor (D, E) (n = 4). MWT = media wall thickness, ED = external diameter. *P<0.05 vs. control, #P<0.05 vs. SuHx. vWF = Von Willebrand Factor.

**Fig 2:**
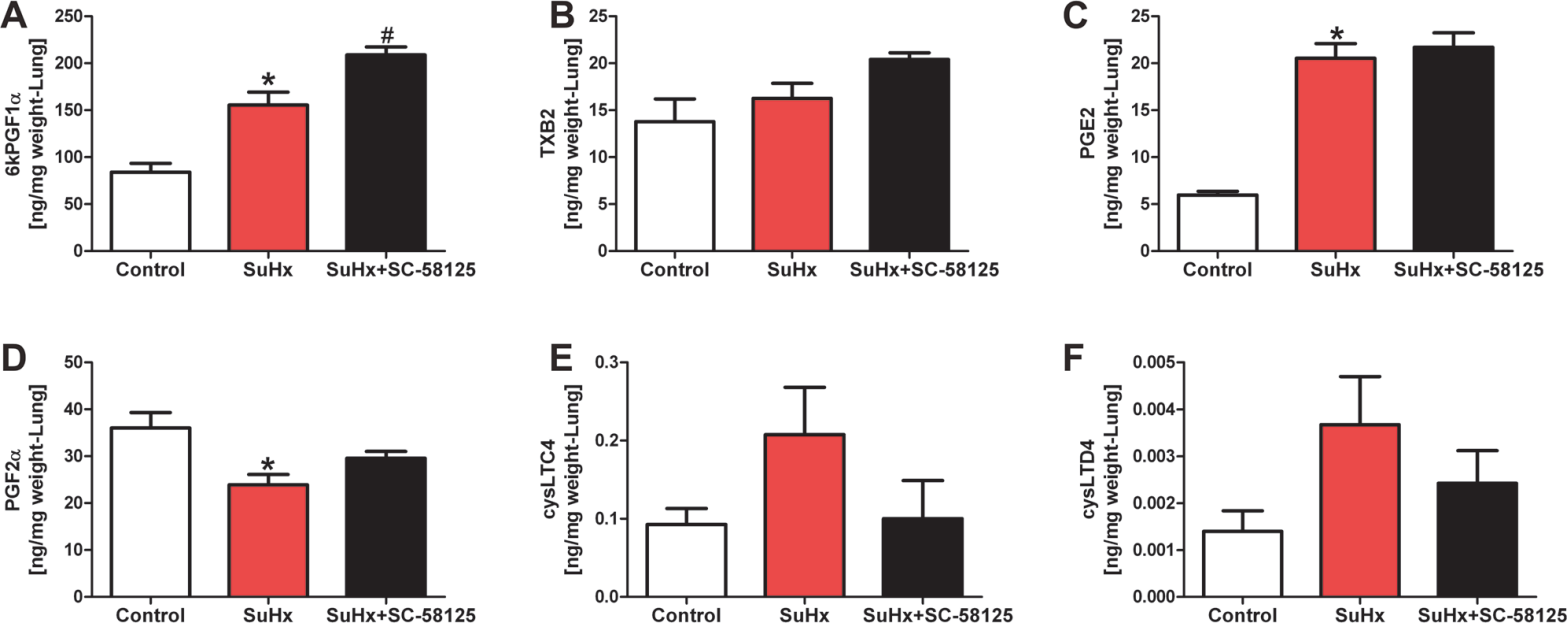
Eicosanoid metabolite concentrations (expressed in ng/mg lung tissue weight) in the lungs. There is an increase in 6-ketoPGF1α (The stable metabolite of prostacyclin), and of PGE2 in the SuHx lung tissues. There was a trend for an increase of the LTC4 and LTD4 levels. Chronic treatment of SuHx rats with the COX-2 inhibitor SC-58125 did not prevent the increase in lung tissue concentration of 6-ketoPGF1α or PGE2 (A, C). The COX-2inhibitor trended to affect the lung tissue increase of the LTC4 and LTD4 (E, F). * P<0.05 vs. control, #P<0.05 vs. SuHx. (n = 4).

**Fig 3:**
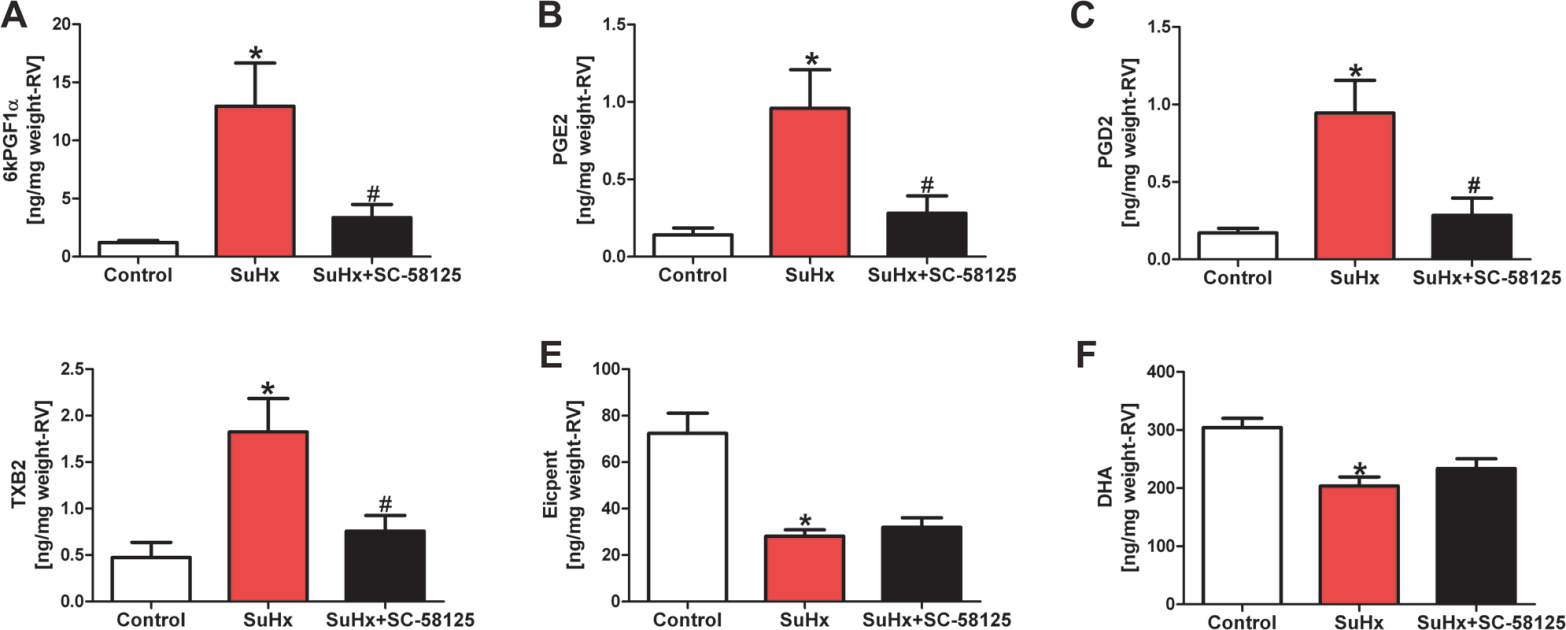
Eicosanoid concentrations in right ventricular tissue samples (expressed as ng/mg right ventricle tissue weight). 6-keto-PGF1α, PGE2, PGD2 and TXB2 levels were elevated in the right ventricle tissues from SuHx rats (A, B, C and D) and this increase was prevented in animals treated with the COX-2 inhibitor. Eicosapentanoic acid (Eicpent) and docosahexanoic acid (DHA) levels were reduced in the RV tissues from the SuHx animals (E, F); treatment with SC-58125 did not affect the levels of eicosapentanoic or docosahexanoic acid levels. * P<0.05 vs. control, #P<0.05 vs. SuHx. (n = 4).

We first analyzed the hemodynamic data to find out whether the COX-2 inhibitor affected right heart pressure and right heart hypertrophy. Dosing of the rats with SC-58125 (10 mg/kg every other day for 21 days) had only a mild effect on the right ventricular systolic pressure at the end of the 3 weeks treatment period, while the degree of RV hypertrophy, muscularization and obliteration of small pulmonary vessels was unaffected by SC-58125 treatment (Fig. 1A-E). To examine whether the chronic COX-2 inhibitor treatment had affected the lung and heart tissue levels of stable eicosanoid metabolites, we measured those in lipid extracts by mass spectroscopy. The combination of Sugen5416 and chronic hypoxia caused an increase in lung tissue eicosanoid metabolites (Fig. 2), but surprisingly the COX-2 inhibitor treatment did not result in a reduction in lung tissue prostacyclin and thromboxane levels of the SuHx rats, while the lung tissue LTC4 and LTD4 levels trended to be lower (but statistical significance was not reached) in the COX-2 inhibitor treated animals (Fig. 2). We also found that the RV tissue levels of 6-keto PGF1α, PGE2, PGD2 and thromboxane B2 were increased in the SuHx animals and that the chronic COX-2 inhibitor treatment had prevented such an increase in the RV tissue levels (Fig. 3). Of note, the lung tissue levels of 6-keto PGF1α, TXB2 and PGE2 were 10, 3 and 5-fold higher respectively when compared to the levels of these metabolites in the RV. We also found a significant reduction of eicosapentaenoic and docosahexaenoic acids in the SuHx RV tissue levels, and that COX-2 inhibitor did not change that reduction (Fig. 3E and F).

### Eicosanoid enzyme proteins are increased in the lungs from SuHx rats

**Fig 4:**
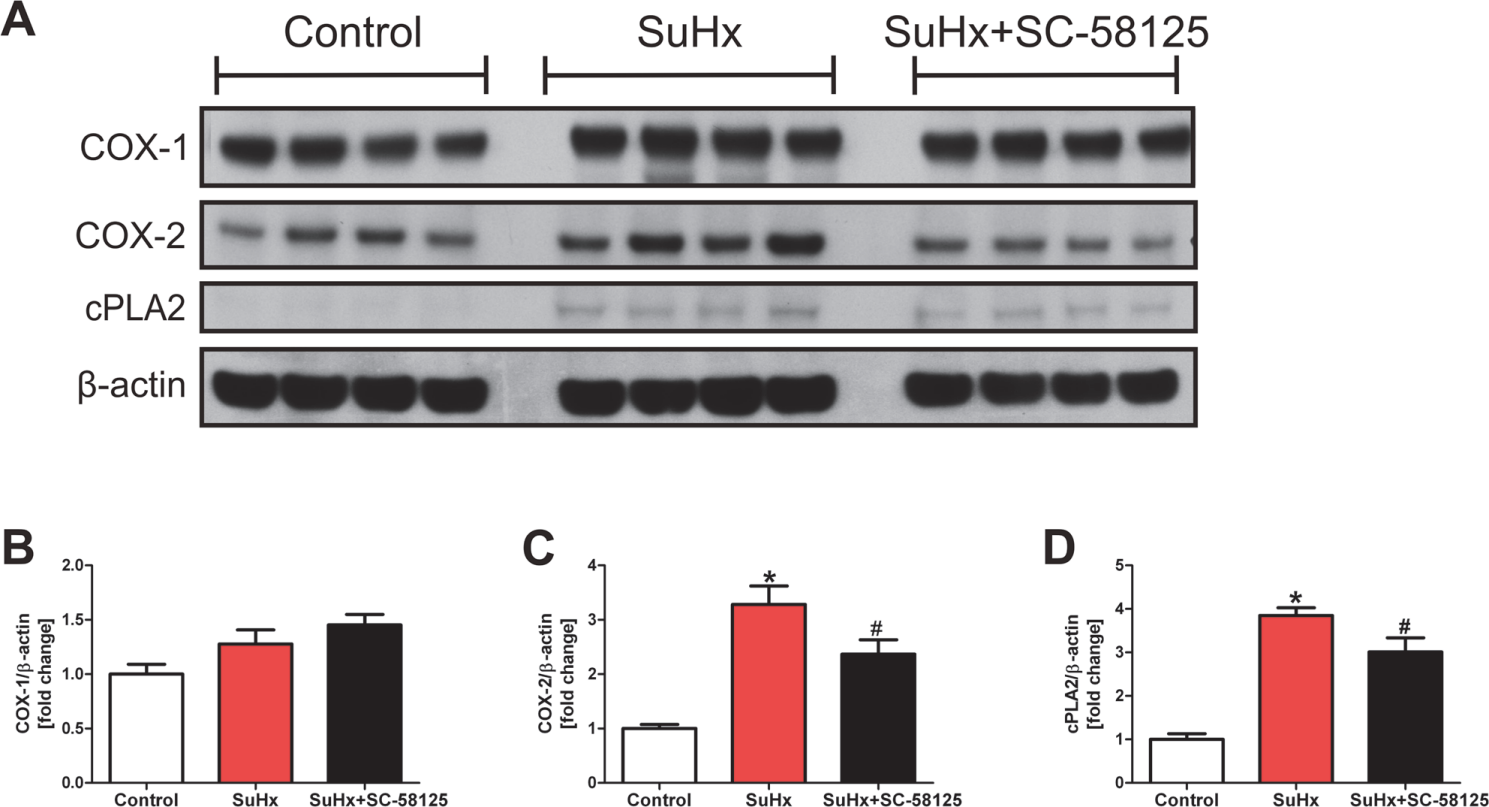
Eicosanoid enzyme protein Western blot data. In lung tissue homogenates there is an increased expression of cPLA2 and COX-2 in the SuHx animals when compared to normal control lung tissues. The overexpression of the COX-2 and cPLA2 proteins is significantly reduced in the lungs from the SuHx animals treated chronically with the COX-2 inhibitor SC-58125 (A). The protein expression is referenced to lung tissue β-actin (B and C). * P<0.05 vs. control, #P<0.05 vs. SuHx. (n = 4).

**Fig 5:**
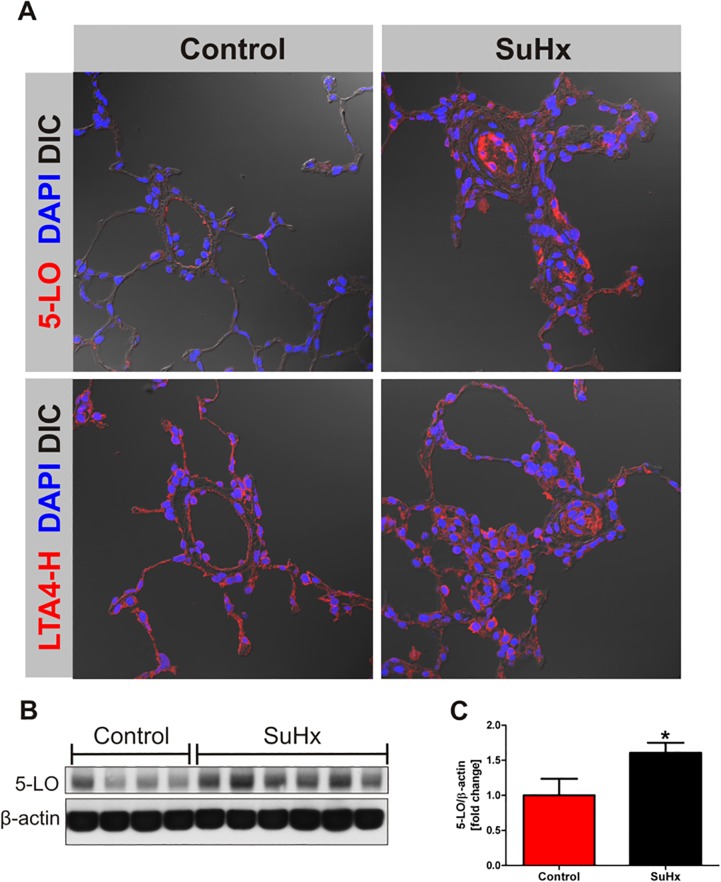
Immunofluorescence for representative tissue samples shows the labeling of lumen-obliterating cells; 5-LO protein expression is increased in the SuHx lungs. Antibodies directed against 5-LO and LTA4 hydrolase were used (B, D), controls (A, C). In control lung tissues there was only sparse labeling of arteriolar endothelial cells for 5-LO, while the LTA4 hydrolase antibody stained alveolar septal cells in addition. In the lungs from the SuHx rats many of the lumen obliterating cells and also perivascular cells were intensely labeled by both antibodies. DAPI = nuclear staining, DIC = differential interference contrast, 5-LO = 5-Lipoxygenase, LTA4-H = Leukotriene A4 hydrolase, magnification 40X. The lung tissue 5-LO protein concentration was increased in the SuHx lungs when analyzed by western blot. (n = 4–6).

In order to investigate whether inhibition of the COX-2 enzyme activity had affected the tissue expression of cytoplasmic phospholipase A2 (cPLA2) and COX-2 proteins in the lungs from the pulmonary hypertensive SuHx rats, we extracted lung tissue protein and subjected the lysates to Western blotting. The lungs from the SuHx rats showed a dramatic increase in the tissue expression of cPLA2 and COX-2 with no significant change of the COX-1 protein expression. Fig. 4 documents the upregulated expression of these enzyme proteins. Fig. 5A illustrates the immunohistochemistry of 5-LO and LTA4 hydrolase which decorate the lumen obliterating lung vascular cells. The increased level of 5-LO in the SuHx lung tissue sample was confirmed by western blotting (Fig. 5B and C).

### Effect of chronic DEC treatment on pulmonary hypertension, right ventricular hypertrophy and lung vessel remodeling

**Fig 6:**
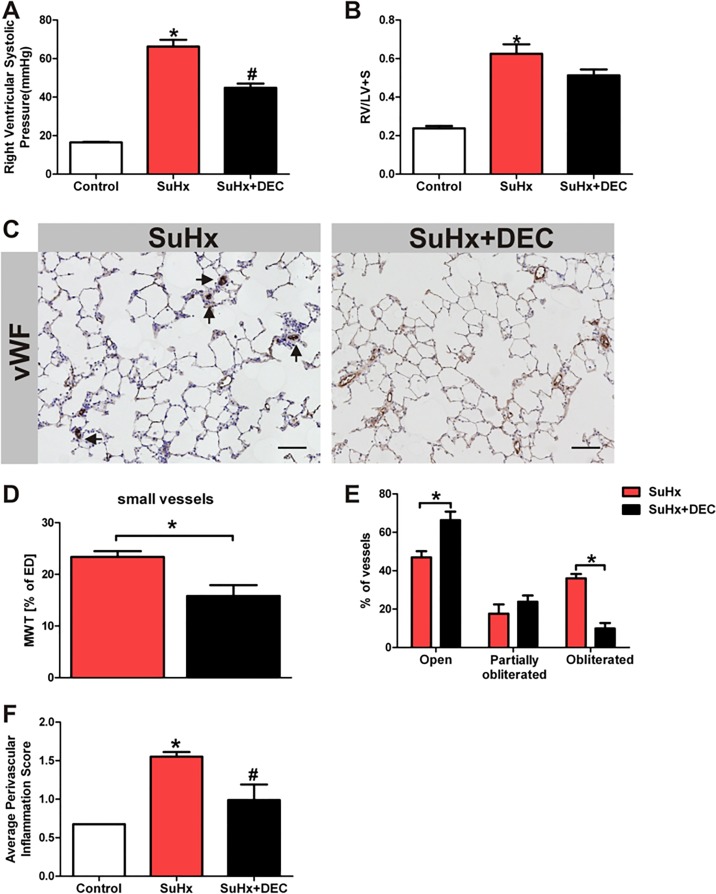
Diethylcarbamazine ameliorated pulmonary angioproliferation & the development of severe PAH. Measurement of the right ventricular systolic pressure (using a Millar catheter) shows a reduction in the RVSP in the SuHx rats following 3 weeks of treatment of the animals with diethylcarbamazine (DEC, 50 mg/kg) (A) (n = 6–8). While there is a reduction of the RVSP in the DEC treated animals there is a trend to reduction in the right ventricular hypertrophy but it did not reach a statistical significance(RV/LV+S) (B) (n = 6–8). (C) Scale bar = 100μm. There is a significant reduction of angioobliteration and muscularization in the lungs from rats treated with DEC (D, E) (n = 6–8). Panel (F) shows that DEC treatment reduced the degree of perivascular cell accumulation (n = 6–8). MWT = media wall thickness, ED = external diameter. * P<0.05 vs. control, #P<0.05 vs. SuHx. vWF = von Willebrand factor

Because of the observed trend towards an elevation of lung tissue levels of LTC4 and LTD4 and the increased expression of the enzyme proteins LTA4 hydrolase and 5-LO in the obliterated vessels of the SuHx lungs (Fig. 5), we treated in additional experiments SuHx rats with diethylcarbamazine (DEC). The 3 week daily treatment of the SuHx rats with DEC partially protected the animals against the pulmonary hypertension which develops as a consequence of the combination of Sugen 5416 injection and exposure of the rats to 3 weeks hypoxia (Fig. 6A). The reduction in right ventricular hypertrophy did not reach statistical significance when SuHx and SuHx rats treated with DEC were compared (Fig. 6B).

In contrast to the treatment of SuHx rats with the COX-2 inhibitor SC-58125, the daily treatment with DEC resulted in a significant reduction in media wall thickness, number of obliterated pulmonary arterioles and the degree of perivascular infiltrates when compared to SuHx animals (Fig. 6C, D, E and F).

### Effect of DEC on Lung and heart eicosanoid metabolites

**Fig 7:**
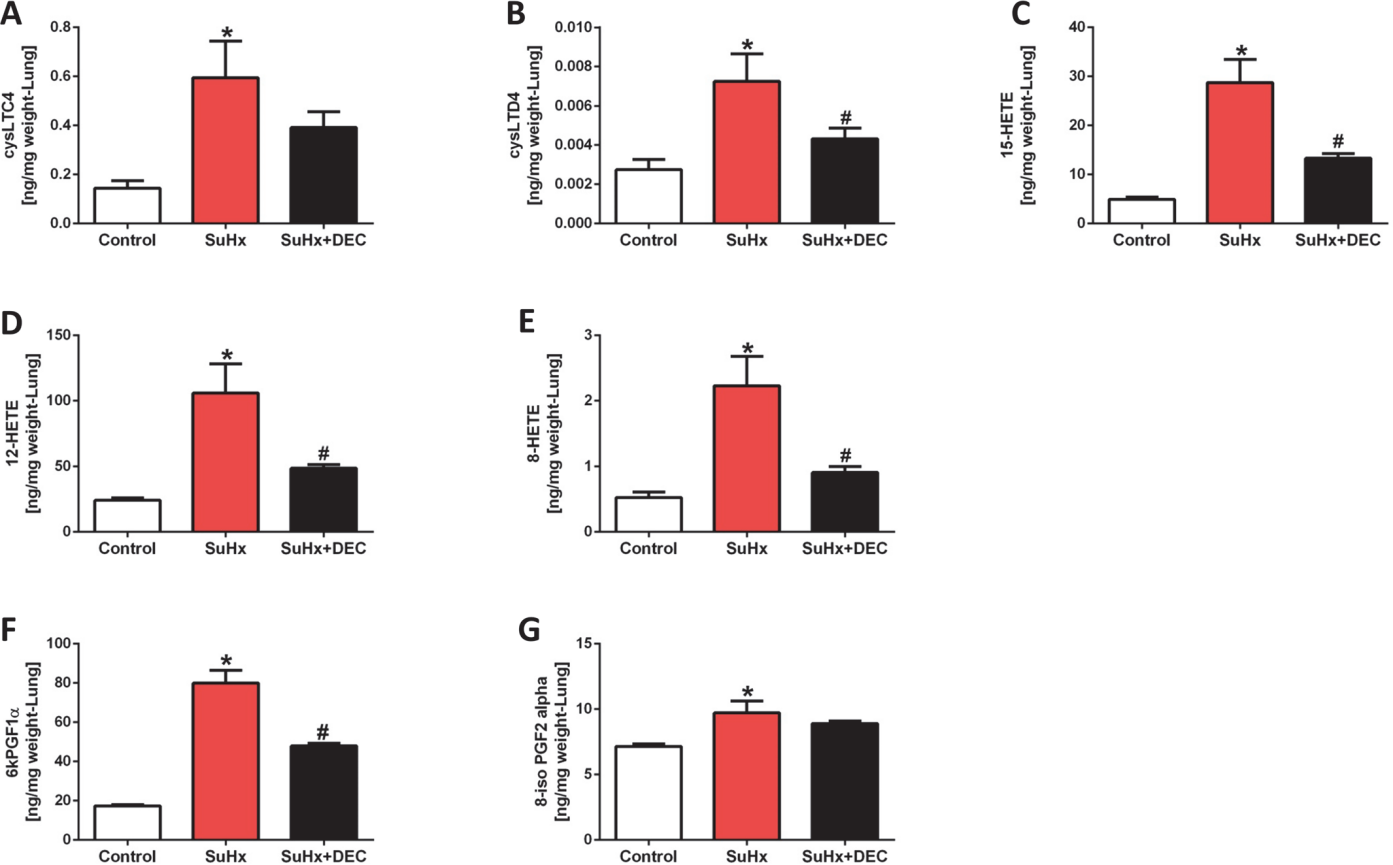
Diethylcarbamazine treatment reduced the increment in lung tissue eicosanoids. Shown are the data obtained from normal control rats, animals treated with SU5416 and exposed to hypoxia for 4 weeks (SuHx) (n = 4) and animals concomitantly treated with diethylcarbamazine (DEC) (n = 6). DEC treatment blunted the increase of LTC4, LTD4, 8, 12, and 15 HETE. In addition, DEC treatment also blunted the increase in 6-keto PGF1α (The stable prostacyclin metabolite) but not the increase in tissue 8-iso PGF2α. * P<0.05 vs. control, #P<0.05 vs. SuHx.

Fig. 7 shows the effect of chronic DEC pretreatment on lung tissue eicosanoids. When lung tissue concentrations of LTC4 and LTD4 were compared between control and SuHx treated animals in this series of experiments we did find a statistically significant increase in the levels of these metabolites (Fig. 7A and B) and not just a trend, as shown in the first series (Fig. 2E and F). DEC treatment trended to reduce the increase of the 5-LO metabolite LTC4 when compared to SuHx rat lungs (Fig. 7A), while the DEC treatment related reduction in the LTD4, 15-HETE, 12-HETE, 8-HETE and 6kPGF1-α levels were significant (Fig. 7B-F). Thus, taken together the data are consistent with DEC affecting the 5-LO activity.

**Fig 8:**
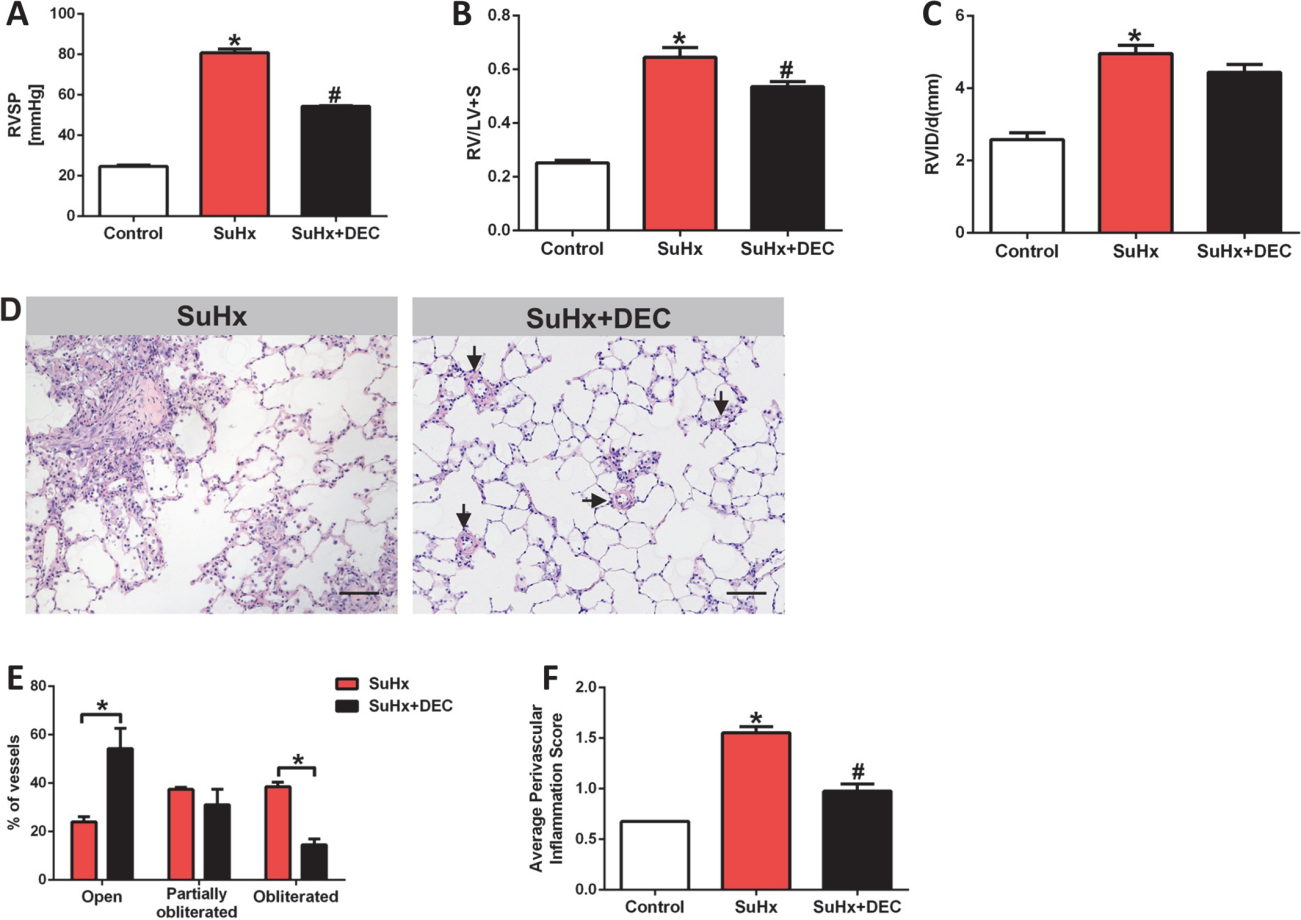
Diethylcarbamazine treatment of animals with established lung vascular disease (intervention trial). (A) Right ventricular systolic pressure (RVSP) in animals subjected to the SuHx protocol (SuHx) (n = 6) and animals treated with diethylcarbamazine (DEC) after pulmonary hypertension had been established (n = 5). There is a treatment related reduction in the RVSP. (B) There is a reduction in the DEC-treated animals’ RV hypertrophy and (C) (n = 6). There is no significant reduction in the right ventricular internal diastolic diameter (RVID/d) (n = 6). (D) Shows representative sections from SuHx rat’s lung treated with a daily dose of 50 mg/kg DEC for 2 weeks, scale bar = 100 μm. (E) Shows that the percentage of open vessels are greater and that there are fewer obliterated vessels after DEC treatment (n = 6). (F) Shows a reduction in the average of perivascular inflammation after treatment with DEC (n = 6). * P<0.05 vs. control, #P<0.05 vs. SuHx.

Fig. 8 shows our results of DEC treatment of established PAH. The rats received DEC injections (5 days/week for 2 weeks) after having been treated with Sugen 5416 and exposed to hypoxia for 4 weeks. Treatment with DEC of animals with established PAH reduced the RVSP and RV hypertrophy (Fig. 8A and B), but the RV internal diameter was not affected significantly (Fig. 8C). Of interest, DEC interventional treatment for 2 weeks did affect the obliteration of the pulmonary arterioles and reduced the degree of perivascular cellular infiltrates (Fig. 8D, E and F).

## Discussion

Pulmonary arterial hypertension is increasingly recognized as a group of lung diseases which are characterized by an inflammatory component and immune system abnormalities [35, 36, 37], however, whether, or to what extent, inflammatory mechanisms are causally involved in the pathogenesis of severe forms of PAH remains unclear. Inflammatory cells are present in many forms of PAH in the lung and levels of various arachidonic acid metabolites are increased in rodent models of chronic hypoxia and monocrotaline-induced PH [38,39]. In human lungs from patients with idiopathic pulmonary arterial hypertension (IPAH) there is increased expression of the enzymes 5-lipoxygenase and FLAP [40], while the expression of the prostacyclin synthase gene and protein is reduced [7,41]. Taken together, these data provide evidence for a disturbed and abnormal lung eicosanoid metabolism in human pulmonary hypertension. Importantly, treatment of patients with severe PAH with prostacyclin has been shown to increase the survival of many patients [7].

While both acute and chronic hypoxia increase the expression of COX-2 in the lung [42], insights regarding the role of COX-2 activity in pulmonary hypertension and pulmonary hypertensive vascular remodeling can now be gained by comparing the reported eicosanoid metabolic profiles and hemodynamic data of monocrotaline and chronic hypoxia-induced PH in COX-2 KO mice [25,26] with the data obtained in the present study in the SuHx model of severe PAH and COX-2 inhibitor treated SuHx rats. While the MCT-treated COX-2 KO mice developed mild PH with an average RVSP of 17mmHg, which was associated with only mild pulmonary artery muscularization, in the chronic hypoxia mouse model of PAH, both RVSP and RV hypertrophy were greater in the COX-2 KO than the WT mice [27]. Although we had, based on the published results, considered that COX-2 inhibitor treatment would worsen the PAH and RV hypertrophy in the SuHx rats, this was not found (Fig. 1). In the context of our present rat model studies, it may be relevant that there are differences in the basal levels of lung tissue eicosanoids between rat and mouse [43].

Of interest, as in the study of the COX-2 KO mice by Seta et al [25], COX-2 inhibition by SC-58125 did not decrease, but instead increased lung tissue 6ketoPGF1α (although Seta et. al. had not measured lung tissue, but BALF levels of 6-keto-PGF1α). In both settings this increase in prostacyclin production can be attributed to a COX-2 inhibitor triggered shift towards COX-1-dependent prostacyclin synthesis. In the study by Seta et al, pulmonary vascular remodeling in MCT-treated mice and lung inflammation were minimal, whereas the remodeling of the lung vessels, and accumulation of inflammatory cell infiltrates in perivascular spaces are very prominent in the SuHx pulmonary hypertensive rats. These vascular inflammatory changes were not affected by the treatment with the COX-2 inhibitor SC-58125 (Fig. 1C). Clearly, our present data show that chronic treatment with the specific COX-2 inhibitor SC-58125 does not prevent the development of severe PAH in this rat model but also that the COX-2 inhibitor did not make the PAH in this model worse.

As we show in Fig. 4, the protein expression of cPLA_2_ and COX-2 is increased in the lung tissue samples from the SuHx animals; these findings are consistent with the notion that lung inflammation is prominent in this model and that inflammation causes the increased levels of eicosanoid metabolites in the lungs. To our surprise, the COX-2 inhibitor SC-58125 treatment did not reduce the increased lung tissue prostaglandin and thromboxane metabolite levels. Ryan et al [44] had reported that SC-58125 induced the production of reactive oxygen species and reduced GSH levels in B-lymphocytes. Whether such an eicosanoid metabolism independent drug related increase in reactive oxygen species contributed to the enhancement of prostacyclin lung tissue levels in our experiments is unclear.

In contrast, our data show that the COX-2 inhibitor, in the dose used, acted as expected and reduced the elevated eicosanoid levels in the RV tissues. When we had previously screened our published RV gene expression database [45] we had found that the PLA_2_ mRNA increased 2-fold and the COX-2 mRNA was increased nearly 3-fold in the RV from the severely pulmonary hypertensive SuHx animals. The previous and present data, taken together, indicate that COX-2 expression and activity are upregulated both in the lungs and the RV tissues, and that the COX-2 inhibitor was able to block the eicosanoid metabolite generation in the stressed RV but not in the inflamed lung (Figs. 2 and 3). One likely explanation for this difference in the drug effect between lung and heart is the large number of inflammatory cells that accumulates in the lungs, but not in the heart, of the SuHx animals and the fold greater eicosanoid metabolite production in the SuHx lungs when compared to that of the SuHx RV. Other RV tissue metabolites which are unchanged by the COX-2 inhibitor treatment are eicosapentaenoic and docosahexaenoic acids. They were found to be significantly reduced in the SuHx RV tissue. These are known to be omega-3 fatty acids that have been reported to prevent pressure-overload induced cardiac fibrosis [46]

Because in older studies the antihelminthic lipoxygenase inhibitor diethylcarbamazine (DEC) had been shown to protect against the development of chronic hypoxia–induced PH in rats [47], we were motivated to examine whether DEC affected the pulmonary vascular obliterations in the SuHx model of severe PAH. An additional rationale for this approach was the increased expression of 5-LO and LTA4 hydrolase proteins in the obliterated vessels’ endothelial cells (Fig. 5) and it is therefore tempting to speculate that the reduction of the lung tissue levels of lipoxygenase metabolites by DEC treatment reflects an overall reduced inflammatory burden of the SuHx lungs (Fig. 8). Whether the reduction in the RVSP and RV hypertrophy can be attributed to inhibition of pulmonary vasoconstriction or due to a reduction of lung inflammation remains to be determined. While DEC treatment reduced RVSP without normalizing it, it did significantly reduce the pulmonary arteriolar lumen obliteration.

As expected, DEC did inhibit 5-LO activity, as can be seen by the reduction in LTD4 metabolite levels; DEC also inhibited the increase in 8-, 12- and 15-lipoxygenase (Fig. 7C-E) and cyclooxygenase (6-keto-PGF1α (Fig. 7F)) products. Thus, DEC is not a specific 5-LO inhibitor, but apparently has a broader spectrum of anti-inflammatory actions. Regardless, the pattern of elevated 8, 12 and 15-HETE together with the increased SuHx lung tissue levels of cysteinyl leukotrienes provided evidence for the activation of lipoxygenase pathways. Because PAH in the SuHx model is associated with activation of lipoxygenase pathways and DEC inhibits this activation, we speculate that the effect of DEC treatment on the angioobliterative component of the SuHx PAH (Fig. 6C, D) is attributable to lipoxygenase inhibition. The DEC treated SuHx rat lungs were characterized by a smaller number of obliterated arterioles. The degree of perivascular inflammation was blunted and the degree of arteriolar muscularization was reduced when compared with untreated SuHx rats (Fig. 6).

Based on these data obtained from this prevention trial we conducted a study where we administered DEC to SuHx rats with established PAH. In this intervention trial, daily DEC treatment for 2 weeks (50 mg/kg) decreased the RVSP (Fig. 8A). Although DEC treatment did not change the right ventricular function significantly as shown by the right ventricular internal diameter (Fig. 8C), the degree of RV hypertrophy was significantly reduced with the interventional treatment (Fig. 8B), while that was not the case in the prevention studies; the reason for this discrepancy is not clear. The number of fully obliterated lung vessels and the degree of perivascular infiltration were reduced (Fig. 8F).Thus, DEC has anti-inflammatory effects in the setting of severe SuHx-induced angioobliterative PAH and the drug is somewhat effective in the treatment of severe pulmonary vascular disease in the SuHx model once established.

## Conclusion

In a model of severe angioobliterative PAH and right heart failure inflammation is prominent and the protein expression of PLA2 and COX-2 is increased in the lung tissue, as are cyclooxygenase and lipoxygenase metabolites. While cyclooxygenase-derived metabolites remained elevated in lungs from the animals treated with a COX-2 inhibitor, this treatment did not worsen the PAH and had no effect on the pulmonary vascular remodeling. However, COX-2 inhibitor treatment of SuHx rats largely prevented the increased production of several cyclooxygenase-dependent eicosanoids in the stressed RV. Both a prevention and intervention treatment trial with the non-specific lipoxygenase inhibitor diethylcarbamazine (DEC) ameliorated the development of pulmonary hypertension, pulmonary inflammation and angioobliterative remodeling. Taken together, our experimental data in this model of severe PAH support the hypothesis that treatments with agents that affect lipoxygenase metabolite production may modify the angioproliferative process. The data of this present investigation complement the data that shows that bestatin, a leukotriene hydrolase inhibitor, prevents the development of PAH and reverses established PAH in immunocompromized T reg cell deficient, athymic rats [48].
